# Correction: Emokpaire et al. Effect of Ru on Deformation Mechanism and Microstructure Evolution of Single-Crystal Superalloys under Medium-Temperature and High-Stress Creep. *Materials* 2023, *16*, 2732

**DOI:** 10.3390/ma17215292

**Published:** 2024-10-31

**Authors:** Stephen Okhiai Emokpaire, Nan Wang, Jide Liu, Chongwei Zhu, Xinguang Wang, Jinguo Li, Yizhou Zhou

**Affiliations:** 1 Shi-Changxu Innovation Center for Advanced Materials, Institute of Metal Research, Chinese Academy of Sciences, Shenyang 110016, China; 2 School of Materials Science and Engineering, University of Science and Technology of China, Shenyang 110016, China; 3 The State Key Laboratory of Rolling and Automation, Northeastern University, Shenyang 110016, China

Following publication, concerns were raised regarding the peer-review process related to the publication of this article [1]. Adhering to our standard procedure, the Editorial Board conducted an investigation which determined that, while the peer-review process does comply with MDPI’s Editorial process policy (https://www.mdpi.com/editorial_process), the contribution of one of the four reviewers does not comply with MDPI’s Guideline for Reviewers (https://www.mdpi.com/reviewers#_bookmark11) or the expectations of the Editorial Board. As a result, the Editorial Board has decided to remove the contribution of Reviewer 2 from the open peer-review record (https://www.mdpi.com/1996-1944/16/7/2732/review_report) and, following discussion with the authors, removed the recommended citations 4 and 12 from this publication as they are irrelevant to the paper’s topic. The order of some references has been adjusted accordingly.

The authors state that the scientific conclusions are unaffected. This correction was approved by the Academic Editor. The original publication has also been updated.
